# Pericardial effusion under nivolumab: case-reports and review of the literature

**DOI:** 10.1186/s40425-019-0760-4

**Published:** 2019-10-18

**Authors:** Saade Anastasia, Mansuet-Lupo Audrey, Arrondeau Jennifer, Thibault Constance, Mirabel Mariana, Goldwasser François, Oudard Stéphane, Weiss Laurence

**Affiliations:** 1 Immunologie Clinique, AP/HP, Hôpital Européen Georges Pompidou, Paris, France; 2 Université Paris Descartes, Sorbonne Paris-Cité, Paris, France; 3 Anatomopathologie, AP/HP, Hôpital Cochin, Paris, France; 4 Oncologie médicale, AP/HP, Hôpital Cochin, Paris, France; 5 Oncologie médicale, AP/HP, Hôpital Européen Georges Pompidou, Paris, France; 6 Unité fonctionnelle de Cardio-oncologie et Prévention, AP/HP, Hôpital Européen Georges Pompidou, Paris, France

**Keywords:** Immune checkpoint inhibitor, Nivolumab, Immune-related adverse event, Pericardial effusion, Tamponade, Cardiotoxicity, Corticotherapy

## Abstract

**Background:**

Nivolumab, a programmed death-1 (PD-1) inhibitor, is an immune checkpoint inhibitor particularly used in the treatment of malignant melanoma, non-small cell lung cancer and renal cell carcinoma. Immune-related adverse events are frequent under immunotherapies. Cardiotoxic side effects, initially thought to be rare, are more often encountered paralleling the expanding use of immune checkpoint blockade. Among them, pericardial effusion and tamponade deserve attention as they may present with unusual symptomatology.

**Case presentation:**

We report three cases of pericardial effusion under nivolumab for lung adenocarcinoma. Two cases of early and late-onset pericardial effusion were symptomatic with tamponade and one case occurred without any symptoms. Pericardiocentesis with pericardial biopsy was performed in symptomatic pericardial effusion followed by the administration of a corticotherapy. Pericardial biopsies showed infiltration of T-lymphocytes, mostly CD4^+^. Nivolumab was stopped in two cases and resumed for one patient. Pericardial effusion evolved positively in all cases with or without treatment.

**Conclusions:**

We review the literature on pericardial effusion under nivolumab to further discuss the hallmarks of pericardial effusion under nivolumab and the management of nivolumab therapy in this situation. In conclusion, pericardial effusion as an immune-related adverse event under nivolumab appears less rare than initially thought and may require particular attention.

## Background

Nivolumab, a programmed death-1 (PD-1) inhibitor, is an immune checkpoint inhibitor (ICI) initially used in the treatment of malignant melanoma, non-small cell lung carcinoma (NSCLC) and renal cell carcinoma. The spectrum of nivolumab is expanding to urothelial cancer, hematologic malignancies with Hodgkin’s disease, and to squamous-cell carcinoma of the head and neck.

Nivolumab, an IgG4 antibody, targets programmed death-1 protein (PD-1) blocking its interaction with PD-1 ligands, programmed death ligand-1 and 2 (PD-L1, 2), to prevent inactivation of previously activated effector T-cells. PD-1 blockade results in the enhancement of host immunity against tumour cells. Such as cytotoxic T-lymphocyte associated protein-4 (CTLA-4), another immune checkpoint, PD-1 is involved in immune tolerance mechanisms preventing the immune system to react against self-antigens. Compared to CTLA-4, PD-1 is believed to inhibit T-cells at later stages of the immune response in peripheral tissues, hence involved in peripheral tolerance [1]. The inhibition of PD-1/PD-L1 is not specific to anti-tumour T-cells, and can affect other PD-1 expressing lymphocytes, including peripheral autoreactive T-cells. Consequently, immune responses against non-targeted organs under ICI, accounting for, so called, immune-related adverse events (IrAEs), are suspected to result from this mechanism [2].

Nivolumab toxicity profile includes a panel of IrAEs from cutaneous rash, colitis, to hepatitis, pneumonitis, and endocrinopathies [3]. Paralleling the expanding use of ICI, IrAEs have gained major interest. Any grade toxicity by Common Terminology Criteria for Adverse Events reaches 60 to 90% patients according to the use of anti-PD-1, anti-CTLA-4, or the combination of both [4]. Interestingly, IrAEs may present with uncommon symptomatology, mimicking progression and even be, life threatening.

Cardiotoxic events are infrequent IrAEs. Among them, cardiac arrest, heart failure, cardiomyopathy, heart block, myocardial fibrosis and myocarditis were documented [5]. Autoimmune myocarditis, sometimes fulminant with fatal outcomes, was reported, early under anti-CTLA-4 antibody, ipilimumab, with an incidence of 0.09% [6]. Incidence and severity increased when anti-PD-1/anti-CTLA-4 were concomitantly used. A pre-existing cardiac pathology or a peripheral artery disease was frequent in the patient’s past medical history [5]. More recently, subclinical acute immune-related myocarditis under nivolumab and ipilimumab was reported with favourable outcomes [7].

Pericardial effusion is a less described IrAEs of ICI. Under ipilimumab, pericardial effusion occurred after 4 cycles [8, 9]. Pathology from pericardial tissue revealed acute inflammation with lymphocyte dominance [8], or lymphocytic pericarditis with reactive mesothelial cells [9], free of malignant cells. Under nivolumab, pericardial effusion appears less documented and may yet be underdiagnosed.

In this paper, we present three cases of pericardial effusion under nivolumab from two university-hospitals over a two-year-period. We next conduct an exhaustive review of the literature on this event to further describe the characteristics of pericardial effusion occurring in oncology patients under nivolumab and discuss the management of pericardial effusion in this context.

## Case presentation

### Case 1

A 58-year-old woman came to the emergency department for acute visual defect and headache. Her medical history included active smoking. A brain magnetic resonance imaging (MRI) revealed multiple pathological cerebral lesions with intracranial hypertension. She was treated with 1 mg/kg/d corticosteroids. A left hilar lung adenocarcinoma (TTF1+) harbouring *KRAS*, *PI3KCa* and *PTEN* mutations was diagnosed, with left adrenal gland and cerebral metastasis. She received *in toto* radiotherapy followed by carboplatin and pemetrexed. Upon 6 cycles, she progressed with hepatic lesions. Meanwhile, steroids were decreased to 40 mg. A second-line treatment by nivolumab 3 mg/kg/2 weeks was initiated.

**Fig. 1:**
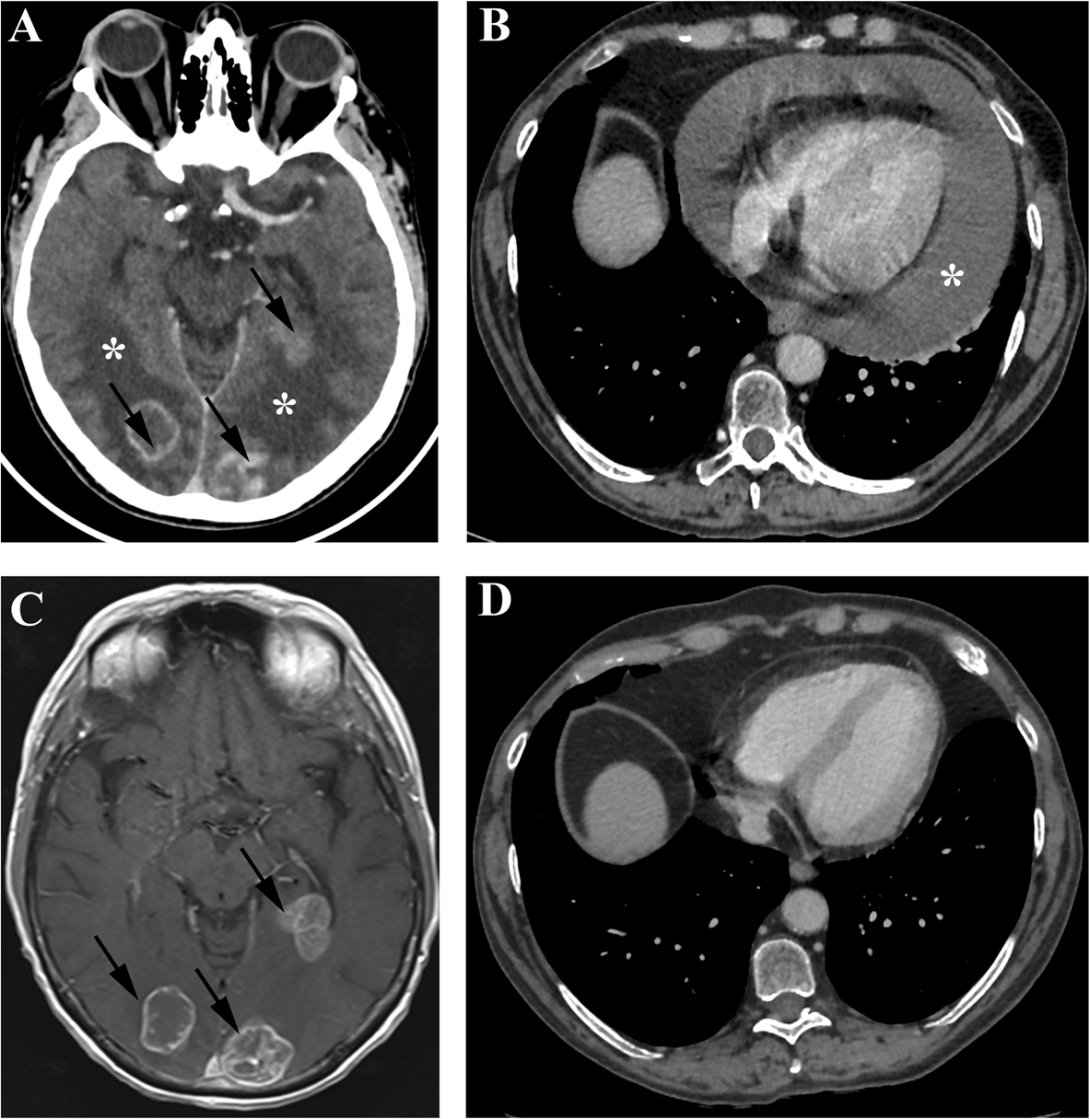
Cerebral and chest imaging of patient 1. **a** Axial cerebral CT section displaying multiple brain lesions (arrows) with perilesional oedema after the 4^th^ infusion of nivolumab. Brain lesions decreased in size while perilesional oedema was significantly increased. **b** Axial chest CT imaging showing cardiomegaly with pericardial effusion (asterisk) after the 4th infusion of nivolumab. Note the absence of radiological evidence of pericardial or pleural cancer involvement, dilatation of the right cavities. **c** Axial gadolinium-enhanced T1-weighted MRI at baseline before the initiation of nivolumab. **d** Axial chest CT imaging at baseline before the initiation of nivolumab

She presented to the emergency department for reoccurrence of headaches with vomiting 12 days after the first dose of nivolumab. As cerebral hypertension was highly suspected, corticotherapy at 1 mg/kg was administered intravenously. Cerebral computed tomography (CT) showed increased perilesional oedema without new lesions. Symptoms evolved favourably, and were controlled under 0.8 mg/kg of prednisone. As she received her fourth infusion, she presented mild dyspnea and cough with clear sputum related to a recent bronchitis in the context of persistent smoking. She was afebrile and had no chest pain. Chest radiography eliminated an interstitial syndrome, stigmatism of pulmonary hypertension and suspected cardiomegaly. Chest CT showed pericardial effusion without radiological evidence of pericardial and pleural cancer involvement, nor dilatation of the right cavities (Fig. 1a and b). Effusion was not visible on the baseline CT (Fig. 1c and d). Imaging also showed disease progression on the left hilar pulmonary nodule, mediastinal nodes, and hepatic lesions, while brain lesions were significantly smaller with increased perilesional oedema (Fig. 1). In the intensive care unit (ICU), echocardiogram revealed massive pericardial effusion close to tamponade. Heart drainage revealed a haemorrhagic and discretely inflammatory liquid. Pericardial biopsy showed small reactive T-lymphocytes predominantly CD4^+^, without cell suspect of malignancy in morphology and immunohistochemistry (TTF1^−^) (Fig. 3a, b, c). After pericardiocentesis and increased doses of corticosteroids, the patient improved rapidly. Immunotherapy was withdrawn after a dedicated multidisciplinary meeting. Subsequent lines by paclitaxel followed by gemcitabine failed to control the disease. The patient died 7 months later from massive proximal pulmonary embolism.

### Case 2

A 65-year-old man, active smoker, was diagnosed with lung adenocarcinoma TTF1+ revealed by a superior vena cava syndrome on mediastinal adenopathy. Tumour was wild-type for *EGFR*, *BRAF*, *KRAS* and *HER2* genes. He initially received 5 cycles of concomitant radiotherapy and chemotherapy by carboplatin and pemetrexed. Progression at 9 months motivated therapy with nivolumab 3 mg/kg/2 weeks. Partial response was observed 3 months later with significant regression of the right adrenal gland metastasis without new lesions. The 4th cycle was complicated with a grade 3 microscopic collagen and lymphocytic colitis histologically confirmed on biopsies. The patient received prednisone followed by entocort. Nivolumab was continued and symptoms resolved under entocort.

**Fig. 2:**
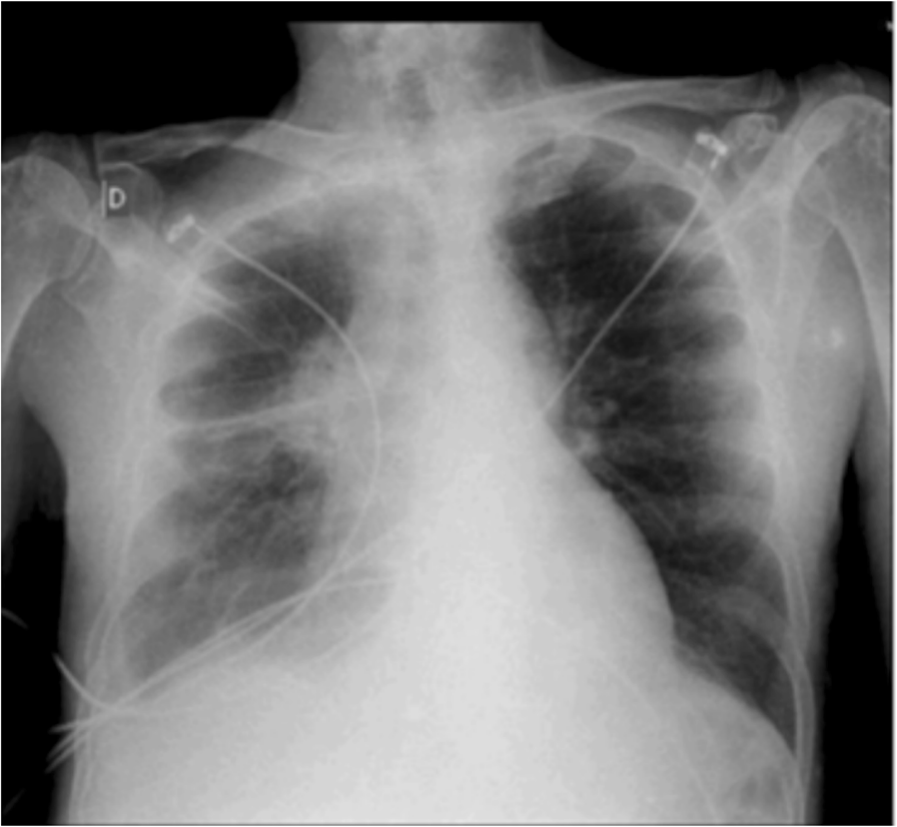
Chest X-ray of patient 2. Chest X-ray performed at in the emergency department showing cardiomegaly. Note the right tumoural lung opacity

**Fig. 3:**
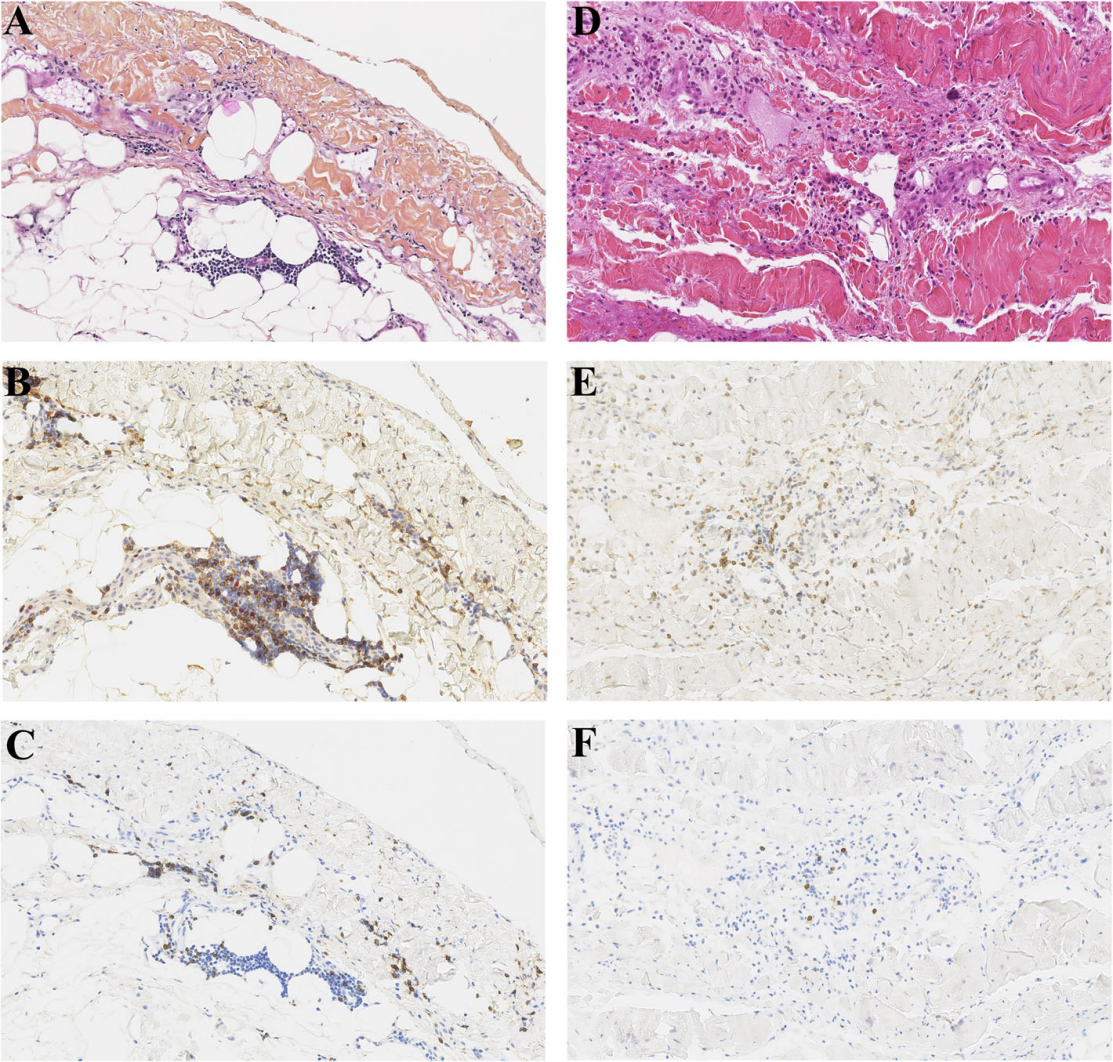
Pathology aspect of non-tumoural pericardial biopsies. Patient 1: Hematoxylin eosin saffron (HES) staining (**a**) (original magnifications × 200) showing reactive lymphocyte infiltrate with more CD4^+^ cells (**b**) than CD8^+^ cells (**c**). Few CD4^+^ cells are FOXP3^+^ (red nuclear staining) (**b**). Patient 2: HES staining (**d**) (original magnifications × 200) showing abundant lymphocyte infiltrate, mostly CD4^+^ (**e**) than CD8^+^ cells (**f**)

He was transferred to the ICU for acute febrile respiratory failure on the 8^th^ day of the 35^th^ infusion of nivolumab. The patient was mechanically ventilated. A probabilistic antibiotherapy was initiated in the hypothesis of a severe pneumonitis. Chest radiography showed a right peri-hilar opacity with cardiomegaly (Fig. 2). A bedside echocardiogram revealed massive pericardial effusion with tamponade. Surgical drainage with pericardial biopsy was performed. Pericardial cytology was haemorrhagic and inflammatory. Microbiology was negative. Pathology from pericardial tissue showed pericardial hyperplasia with T-lymphocyte infiltrate, mostly CD4^+^ (Fig. 3d, e, f), without lesion suspected of malignancy, confirmed by immunohistochemistry (TTF1^−^).

Troponin was normal. Flu was negative. Legionella and pneumococcus urinary antigens were negative. Microbiology from the bronchoalveolar lavage was negative. Exploration for autoimmune diseases was negative (complement, rheumatoid factor, anti-nuclear and anti-dsDNA antibodies, anti-neutrophil cytoplasmic antibodies, and myositis-associated auto-antibodies). CT-imaging showed no sign of progression, a reinforcing argument against neoplastic pericarditis. The patient was successfully treated with corticosteroids for 3 months. As immune pericarditis was highly suspected, immunotherapy was stopped, and resumed 16 months later. The patient remains progression-free without recurrence of pericarditis at 6 months of treatment.

### Case 3

A 55-year-old woman, active smoker, diagnosed with a stage IIIB lung adenocarcinoma TTF1^+^ and ALK^−^, was treated by cisplatin and vinorelbine with radiotherapy. Progression of the pulmonary mass with the appearance of a new contralateral lesion and bone metastasis motivated the introduction of nivolumab. As the patient had a history of ischemic cardiopathy, she had frequent cardiac monitoring. Pericardium was normal. After the third cycle, she developed grade 2 diarrhea. CT evaluation showed disease progression, colitis and pericardial effusion. Echocardiogram confirmed the pericardial effusion of approximately 13 mm around the left ventricle without hemodynamic compromise. Drainage was not performed, neither pericardial biopsy. Nivolumab was stopped for disease progression. Diarrhea resolved spontaneously. Pericardial effusion regressed spontaneously 1 month after the cessation of nivolumab. Cancer progressed with cerebellar and pleural metastasis despite the introduction of a third line by pemetrexed. Altered condition led to the decision of palliative care.

## Discussion and conclusions

Under nivolumab, pericardial effusion and cardiac tamponade were observed with an incidence of 0.69% in a phase III trial comparing nivolumab and docetaxel in 287 patients with NSCLC [Checkmate 057] [10]. Pericardiocentesis for pericardial effusion was reported to be uncommon in nivolumab-treated patients (10/1798), as recently highlighted in a retrospective study performed over a two-year-period [11].

Despite the low incidence of pericardial effusion under nivolumab in these studies, 13 cases have been reported in the literature since 2016 [12–21]. We report on 3 new cases of early and late-onset pericardial effusion in patients with NSCLC treated with nivolumab. The baseline characteristics of all patients (*n* = 16) are presented in Table 1. Patients were predominantly smoker man of 63 ± 7 years old. Two patients had previous autoimmune disease. All patients were treated for metastatic lung cancer, mostly adenocarcinoma. This observation may not result from a particular link with pulmonary neoplasia, as nivolumab is still mostly used in this context. Pericardial effusion is described in other types of cancer with the expanding indications approved by the Food and Drug Association for nivolumab [11]. All patients had already received at least one line of chemotherapy, 63% had prior thoracic irradiation and 50% presented pericardial effusion from mild to moderate at relapse. Pericardial effusion occurred at any time from the initiation of nivolumab, approximately after 5 cycles, but could be of early (1 cycle) [12, 15] or late-onset (35 cycles) [20]. Most common symptoms at diagnosis were dyspnea followed by tachycardia and chest pain. Shock was present in 31% of the cases. Tamponade was detected in 81% at initial presentation. Interestingly, subclinical, and even asymptomatic, pericardial effusion was described [ [17], Case 3]. Consequently, the incidence of immune-related pericarditis might be higher than reported. Routine echocardiographic monitoring may be helpful to assess the true incidence of immune-related pericarditis.

**Table 1:** Patients with pericardial effusion under nivolumab: demographic and clinical characteristics

	All patients (*n* = 16)		Previous cases (*n* = 13)		This work (*n* = 3)	
	n	%	n	%	n	
Patient’s characteristics						
Male	12	(75)	11	(86)	1	
Age (years ± SD)	63	±7	64	±7	59	±4
Smoker	11	(69)	8	(62)	3	
Type of tumour						
S-NSCLC	2	(13)	2	(15)	0	
A-NSCLC	13	(81)	10	(77)	3	
SCLC	1	(6)	1	(8)	0	
Stage						
IIIb	4	(25)	4	(31)	0	
IV	12	(75)	9	(69)	3	
Malignant pericardial effusion	8	(50)	6	(46)	2	
n-line therapy						
2	10	(63)	7	(54)	3	
3	3	(19)	3	(23)	0	
> 3	2	(13)	2	(15)	0	
Previous therapeutics						
Thoracic irradiation	10	(63)	7	(54)	3	
Cisplatin	6	(38)	5	(38)	1	
Carboplatin	11	(69)	9	(69)	2	
Paclitaxel	3	(19)	3	(23)	0	
Pemetrexed	9	(56)	7	(54)	2	
Etoposide	2	(13)	2	(15)	0	
Tyrosine kinase inhibitors	3	(19)	3	(23)	0	
Bevacizumab	3	(19)	3	(23)	0	
Others^a^	5	(31)	4	(31)	1	
Pericardial effusion						
Time of onset (cycles, median (range))	5	(1–35)	5	(1–24)	6	(4–35)
Initial symptoms						
Dyspnea	11	(69)	9	(69)	2	
Chest pain	3	(19)	3	(23)	0	
Shock	5	(31)	4	(31)	1	
Respiratory failure	4	(25)	3	(23)	1	
Tachycardia	5	(31)	5	(38)	0	
Tamponade	13	(81)	11	(85)	2	
Asymptomatic	2	(13)	1	(8)	1	
Others^b^	3	(19)	2	(15)	1	
Treatment						
Pericardiocentesis	11	(69)	10	(77)	1	
Pericardial window	5	(31)	4	(31)	1	
Surgical drainage	2	(13)	1	(8)	1	
Corticosteroids	7	(44)	5	(38)	2	
Colchicine	2	(13)	1	(8)	1	
Nivolumab use						
Stopped	10	(63)	8	(62)	2	
Continued	2	(13)	2	(15)	0	
Stopped and Resumed	4	(25)	3	(23)	1	
Outcome						
Progression	1	(6)	0	(0)	1	
Pseudoprogression	8	(50)	7	(54)	1	
Resolution of pericardial effusion	12	(75)	9	(69)	3	
Other IrAEs	7	(44)	5	(38)	2	
Recurrent pericardial effusion	3	(19)	3	(23)	0	
Hypothyroiditis	2	(13)	2	(15)	0	
Colitis	2	(13)	0	(0)	2	
Pneumonitis	1	(6)	1	(8)	0	
Pericardial fluid cytology						
Malignant cells	6	(38)	6	(46)	0	
Leukocytes	8	(50)	6	(46)	2	
Serosanguinous	7	(44)	6	(46)	1	
Pericardial biopsy						
Malignant cells	0	(0)	0	(0)	0	
Lymphocytes	4	(25)	3	(23)	1	
Atypical cells	2	(13)	1	(8)	1	
Inflammation	5	(31)	5	(38)	0	
Fibrosis	4	(25)	4	(31)	0	
Fibrinous	3	(19)	3	(23)	0	
Mesothelial hyperplasia	2	(13)	1	(8)	1	

Data are given as absolute value with percentage for all patients (*n* = 16), for patients from case reports reported in the literature (*n* = 13) and from our 3 cases

^a^Topotecan, Everolimus, Temozolamide, Docetaxel, Gemcitabine, Vinorelbine, S-1

^b^Caughing (1), Fever (1), cardiac arrest (1)

Pericardial effusion related to pseudoprogression was reported in 8/16 (50%) patients. Patients with pseudoprogression often had prior pericardial effusion (75%) and malignant cells were found in the pericardial fluid (75%). Pseudoprogression is described as a transient increase in tumour size followed by regression, or the appearance of new lesions in the presence of a response of other target lesions [17, 22]. The diagnosis of pseudoprogression requires a longitudinal follow-up demonstrating a delayed tumour response, while the ICI is not resumed. Indeed, at the time of diagnosis, it is difficult to differentiate whether pericardial effusion results from cardiac tumour progression, from an immune-mediated pericarditis, from an infection or from the exacerbation of a pre-existing cardiac disease under nivolumab. Malignant pericardial effusion reached 1.6 to 20% in historical autopsy series. In the case of initial malignant pericardial effusion, treatment with nivolumab seemed to favour recurrent pericarditis [12, 16, 18]. Thus, subsequent clinical course as well as microbiological and anatomopathological analyses may help to the differential diagnoses. Myocarditis was not detected in reported cases. In our work, myocarditis was ruled out based on clinical, electrocardiogram, biological and echocardiogram findings. Investigations for autoimmune diseases were negative.

Cytology revealed malignant cells in 6/16 (38%) patients, leukocytes in half the cases. Microbiology was negative in all pericardial fluid.

Pathology from pericardial biopsies was free from malignant cells, revealed mild or extensive fibrosis with non-specific inflammation [13, 16, 19, 20], consisting in lymphocyte infiltration [13, 20], as herein. Interestingly, we identified predominant infiltration by CD4^+^ compared to CD8^+^ lymphocytes (Case 1, 2), with cells expressing both CD4^+^ and FOXP3^+^ (Case 1, Fig. 3), while others reported equal distribution of CD4^+^ and CD8^+^ lymphocyte infiltration [13], with no data reported on FOXP3 expression.

In the context of autoimmune/inflammatory pericarditis, the expression of FOXP3 on infiltrating CD4^+^ T-lymphocytes may result from T-cell activation, as activated CD4^+^ T-lymphocytes transiently express FOXP3 [23]. Conversely, FOXP3 is stably expressed in regulatory T-cells. On the contrary, histology from immune-related myocarditis, was described as CD8^+^-mediated [24]. Pathological lesions from pericarditis, myocarditis, as well as that of autoimmune hepatitis, differ, indicating that they may involve different mechanisms. Autoimmune pericarditis was shown to relate to type I interferon response [25]. Viruses, persistent inflammation with the secretion of interleukin 1-β, were also suggested to be upstream inducers.

Mechanisms driving cardiac IrAEs are still unclear and are believed to result from disturbances in immune checkpoint functions in maintaining immunological homeostasis. Actually, PD-1 plays a role in maintaining self-tolerance. It remains unknown whether autoantibodies or autoreactive T-cells are responsible for IrAEs. Most likely, IrAEs results from the interplay of both humoral and cellular immune responses. Activation of autoreactive T-cells leads to the production of auto-antibodies by autoreactive B-lymphocytes via CD4^+^ T-cells. Moreover, cytotoxic events driven by the interaction between autoantibodies and complement, and CD8^+^ autoreactive T-cells are involved. These mechanisms account for T-cell infiltration in organ biopsies. Other partners are not to be forgotten as inflammatory cytokines, and innate immune cells [3]. Activation of the immune system may be in agreement with the extent to which patients with IrAEs present good responses to nivolumab suggested by a parallel activation of anti-tumour T-cells and autoreactive T-cells leading to inflammatory side effects of non-targeted organs. Interestingly, in patients with cardiac IrAEs, occurrence of other IrAEs was frequent, 63% under ipilimumab [5] and 44% under nivolumab (Table 1).

Pericardial effusion required pericardiocentesis in 11/16 (69%) patients, while pericardial window was needed in 31%. Corticosteroids were administered in less than half of the cases (Table 1). Pericardial effusion evolved favourably in 75%, except for one patient who died from cardiac arrest [13] and 3 patients who developed recurrent pericardial effusion (Table 1).

In half the cases, nivolumab was stopped despite the absence of progression (94%). Nivolumab was continued in 2 patients: one presented a complete response [18], while the other showed partial response followed by a relapse 5 cycles later [14]. Treatment was resumed in 25%, without recurrence of pericardial effusion [ [12, 13, 15], Case 2].

These observations open up new insights concerning the management of IrAEs. Despite that the treatment of IrAEs is based on corticotherapy, in the real life, few patients received this treatment. Corticosteroids were reported to induce rapid and complete resolution of IrAEs, provided that IrAEs were promptly diagnosed and managed. Other treatments were reported as TNF-α monoclonal antibodies or mycophenolate mofetil, with positive outcomes [26]. The consensus from the Society for immunotherapy of Cancer recommends discontinuing ICI permanently after life-threatening IrAEs [27]. The definitive interruption of nivolumab may be challenged, as continuation or resumption of nivolumab after pericardial effusion may be beneficial for the patient, as observed in case 2. The decision to reintroduce nivolumab should be discussed in a multidisciplinary meeting and is based on the benefit-risk ratio and on whether alternative oncologic treatments are available. In our opinion, according to the previous and present case reports, the occurrence of pericardial effusion does not contra-indicate ICI after resolution. Additionally, we recommend routine echocardiogram monitoring for all patients.

The diagnosis of pericardial effusion or cardiac tamponade related to nivolumab remains a diagnosis of exclusion. This hypothesis relies on a series of arguments: i. temporal relationship between the onset of symptoms and nivolumab initiation, ii. absence of another identified cause (heart dysfunction, auto-immune disease, infection …), iii. Rapid resolution under corticotherapy, iv. response of targeted lesions to nivolumab, and v. presence of another IrAE.

As a wide spectrum of life-threatening IrAEs may occur under immunotherapies, clinical vigilance is required with close follow-up. Banal symptoms such as dyspnea should not be neglected and should lead to cardiac examination, as they may be the only symptom announcing cardiac tamponade. Additionally, care should be taken for patients with medical history of pericardial effusion, and previous thoracic irradiation.

## Data Availability

All data generated or analysed during this study are included in this published article. Data sharing is not applicable to this article as no datasets were generated or analysed during the current study.

## Abbreviations

CD: Cluster of differentiation
CTLA-4: Cytotoxic T-lymphocyte associated protein-4
HES: Hematoxylin eosin saffron
ICI: Immune checkpoint inhibitor
IrAE: Immune-related adverse event
MRI: Magnetic resonance imaging
PD-1: Programmed death-1
PD-L1: Programmed death-ligand 1
TTF1: Thyroid transcription factor 1

## References

[1] Boussiotis VA (2016). Molecular and biochemical aspects of the PD-1 checkpoint pathway. N Engl J Med.

[2] Michot JM, Bigenwald C, Champiat S, Collins M, Carbonnel F, Postel-Vinay S (2016). Immune-related adverse events with immune checkpoint blockade: a comprehensive review. Eur J Cancer Oxf Engl 1990.

[3] Postow MA, Sidlow R, Hellmann MD (2018). Immune-Related Adverse Events Associated with Immune Checkpoint Blockade. N Engl J Med.

[4] Larkin J, Chiarion-Sileni V, Gonzalez R, Grob JJ, Cowey CL, Lao CD (2015). Combined Nivolumab and Ipilimumab or Monotherapy in untreated melanoma. N Engl J Med.

[5] Heinzerling L, Ott PA, Hodi FS, Husain AN, Tajmir-Riahi A, Tawbi H (2016). Cardiotoxicity associated with CTLA4 and PD1 blocking immunotherapy. J Immunother Cancer..

[6] Johnson DB, Balko JM, Compton ML, Chalkias S, Gorham J, Xu Y (2016). Fulminant myocarditis with combination immune checkpoint blockade. N Engl J Med.

[7] Thibault C, Vano Y, Soulat G, Mirabel M. Immune checkpoint inhibitors myocarditis: not all cases are clinically patent. Eur Heart J. 2018;10. 10.1093/eurheartj/ehy485 30107497

[8] Yun S, Vincelette ND, Mansour I, Hariri D, Motamed S (2015). Late onset ipilimumab-induced pericarditis and pericardial effusion: a rare but life threatening complication. Case Rep Oncol Med.

[9] Dasanu CA, Jen T, Skulski R (2017). Late-onset pericardial tamponade, bilateral pleural effusions and recurrent immune monoarthritis induced by ipilimumab use for metastatic melanoma. J Oncol Pharm Pract Off Publ Int Soc Oncol Pharm Pract.

[10] Borghaei H, Paz-Ares L, Horn L, Spigel DR, Steins M, Ready NE (2015). Nivolumab versus Docetaxel in advanced nonsquamous non-small-cell lung Cancer. N Engl J Med.

[11] Palaskas N, Morgan J, Daigle T, Banchs J, Durand J-B, Hong D (2019). Targeted Cancer therapies with pericardial effusions requiring Pericardiocentesis focusing on immune checkpoint inhibitors. Am J Cardiol.

[12] Asai M, Kato Y, Kawai S, Watanabe K, Yomota M, Okuma Y (2019). Management of cardiac tamponade during nivolumab of lung cancer with intrapericardial bleomycin: case report. Immunotherapy..

[13] Altan M, Toki MI, Gettinger SN, Carvajal-Hausdorf DE, Zugazagoitia J, Sinard JH (2019). Immune checkpoint inhibitor-associated pericarditis. J Thorac Oncol Off Publ Int Assoc Study Lung Cancer.

[14] Yamasaki M, Daido W, Saito N, Funaishi K, Okada T, Kawamoto K (2019). Pericardial effusion with Tamponade in lung Cancer patients during treatment with Nivolumab: a report of two cases. Front Oncol.

[15] Shaheen S, Mirshahidi H, Nagaraj G, Hsueh C-T. Conservative management of nivolumab-induced pericardial effusion: a case report and review of literature. Exp Hematol Oncol [Internet]. 2018 May 8;7. Available from: https://www.ncbi.nlm.nih.gov/pmc/articles/PMC5941729/10.1186/s40164-018-0104-y PMC5941729 29761026

[16] Vittorio A, Sharma R, Siejka D, Bhattarai K, Hardikar A. Recurrent pericardial effusion while receiving Nivolumab for metastatic lung adenocarcinoma: case report and review of the literature. Clin Lung Cancer. 2018;26. 10.1016/j.cllc.2018.05.010 29937384

[17] Zarogoulidis P, Chinelis P, Athanasiadou A, Tsiouda T, Trakada G, Kallianos A (2017). Possible adverse effects of immunotherapy in non-small cell lung cancer; treatment and follow-up of three cases. Respir Med Case Rep.

[18] Kolla BC, Patel MR (2016). Recurrent pleural effusions and cardiac tamponade as possible manifestations of pseudoprogression associated with nivolumab therapy- a report of two cases. J Immunother Cancer.

[19] Nesfeder J, Elsensohn AN, Thind M, Lennon J, Domsky S (2016). Pericardial effusion with tamponade physiology induced by nivolumab. Int J Cardiol.

[20] de Almeida DVP, Gomes JR, Haddad FJ, Buzaid AC. Immune-mediated Pericarditis With Pericardial Tamponade During Nivolumab Therapy. J Immunother [Internet]. 2018 Feb 16 [cited 2018 Apr 12];Publish Ahead of Print. Available from: https://insights-ovid-com.hellebore.biusante.parisdescartes.fr/pubmed?pmid=29461982 10.1097/CJI.0000000000000217 29461982

[21] Kushnir I, Wolf I (2016). Nivolumab-induced pericardial Tamponade: a case report and discussion. Cardiology..

[22] Chiou VL, Burotto M (2015). Pseudoprogression and immune-related response in solid tumors. J Clin Oncol Off J Am Soc Clin Oncol.

[23] Wang J, Ioan-Facsinay A, van der Voort EIH, Huizinga TWJ, Toes REM (2007). Transient expression of FOXP3 in human activated nonregulatory CD4+ T cells. Eur J Immunol.

[24] Mahmood SS, Fradley MG, Cohen JV, Nohria A, Reynolds KL, Heinzerling LM (2018). Myocarditis in patients treated with immune checkpoint inhibitors. J Am Coll Cardiol.

[25] Xu B, Harb SC, Cremer PC (2017). New insights into pericarditis: mechanisms of injury and therapeutic targets. Curr Cardiol Rep.

[26] Villadolid J, Amin A (2015). Immune checkpoint inhibitors in clinical practice: update on management of immune-related toxicities. Transl Lung Cancer Res.

[27] Puzanov I, Diab A, Abdallah K, Bingham CO, Brogdon C, Dadu R (2017). Managing toxicities associated with immune checkpoint inhibitors: consensus recommendations from the Society for Immunotherapy of Cancer (SITC) toxicity management working group. J Immunother Cancer..

