# Correction to: Pericardial effusion under nivolumab: case-reports and review of the literature

**DOI:** 10.1186/s40425-019-0822-7

**Published:** 2019-12-02

**Authors:** Anastasia Saade, Audrey Mansuet-Lupo, Jennifer Arrondeau, Constance Thibault, Mariana Mirabel, François Goldwasser, Stéphane Oudard, Laurence Weiss

**Affiliations:** 1 Immunologie Clinique, AP/HP, Hôpital Européen Georges Pompidou, Paris, France; 2 Université Paris Descartes, Sorbonne Paris-Cité, Paris, France; 3 Anatomopathologie, AP/HP, Hôpital Cochin, Paris, France; 4 Oncologie médicale, AP/HP, Hôpital Cochin, Paris, France; 5 Oncologie médicale, AP/HP, Hôpital Européen Georges Pompidou, Paris, France; 6 Unité fonctionnelle de Cardio-oncologie et Prévention, AP/HP, Hôpital Européen Georges Pompidou, Paris, France

**Correction to: J ImmunoTher Cancer (2019) 7:266**

**https://doi.org/10.1186/s40425-019-0760-4**

Following publication of the original article [1], the authors reported that authors’ given and family names haven been incorrectly tagged.

Below the authors’ name correctly tagged.

Given name: Anastasia

Family name: Saade

Given name: Audrey

Family name: Mansuet-Lupo

Given name: Jennifer

Family name: Arrondeau

Given name: Constance

Family name: Thibault

Given name: Mariana

Family name: Mirabel

Given name: François

Family name: Goldwasser

Given name: Stéphane

Family name: Oudard

Given name: Laurence

Family name: Weiss

The corrections have been implemented in the original article as well.
